# Overexpression of LIM Homeodomain Gene *Arrowhead* Induces Pleiotropic Developmental Alterations in the Silkworm, *Bombyx mori*

**DOI:** 10.3390/biology14091248

**Published:** 2025-09-11

**Authors:** Nur Fazleen Binti Idris, Chunping Hou, Zhongyi Liu, Lulu Liu, Chunyan Yang, Zongmeng Yang, Hai Hu, Fangyin Dai, Xiaoling Tong

**Affiliations:** 1 State Key Laboratory of Resource Insects, Institute of Sericulture and Systems Biology, Southwest University, Chongqing 400715, China; nurfazleenidris@gmail.com (N.F.B.I.); hcp235699@163.com (C.H.); zyliuswu@163.com (Z.L.); swuliululu@email.swu.edu.cn (L.L.); swu112023408002460@email.swu.edu.cn (C.Y.); lebronzm@email.swu.edu.cn (Z.Y.); huhaiswu@163.com (H.H.); 2 Key Laboratory of Sericultural Biology and Genetic Breeding, Ministry of Agriculture and Rural Affairs, College of Sericulture, Textile and Biomass Sciences, Southwest University, Chongqing 400715, China

**Keywords:** silkworm, *Bombyx mori*, *Arrowhead* gene, development, LIM homeodomain, overexpression, pleiotropic

## Abstract

LIM homeodomain (LIM-HD) is a versatile family of transcription factors that act as master regulators in various developmental processes of eukaryotes, and one of the LIM-HD encoded genes is the *arrowhead* (*AWH*). In silkworm *Bombyx mori*, the *Arrowhead* gene (*BmAWH*) functions as a key component activating all three fibroin genes in the silk glands of *B. mori*, but the potential pleiotropic effects of *BmAWH* on various tissues of the silkworm is yet to be discovered. The objective of this study is to investigate the functional role of a *BmAWH* gene in the *B. mori* (Dazao) developmental process, using the piggyBac-based transgene technique. The size of transgenic line silk glands have become smaller, resulting in the reduction in whole cocoon weight, cocoon shell weight, and cocoon–shell ratio. Overexpression of *BmAWH* has induced significant changes in juvenile hormone levels in female larvae at the fifth instar larval stage. Female reproductive defects (reduction in fecundity rate, abnormal egg morphology) were observed. In addition, transgenic line larvae exhibit the complete disappearance of larval body patterns and color (melanin pigmentation). Since the LIM-HD protein functions to orchestrate complex developmental programs, this study may shed light on evolutionary adaptations and the divergence of insect gene functions.

## Simple Summary

The *Bombyx mori Arrowhead* gene (*BmAWH*) is one of the gene encodes for LIM homeodomain (LIM-HD) family genes. Previous studies have systematically assessed the function of *BmAWH* as a key component activating all three fibroin genes of silkworm silk glands. However, how *BmAWH* overexpression affects the other tissues’ development remained unexplored. This study revealed that *BmAWH* plays multiple regulatory roles in silkworm development, as demonstrated by the piggyBac-based transgene overexpression of *BmAWH* in silkworm *B. mori* as an animal model. High expression levels of *BmAWH* affect the silk gland size and cocoon traits, reproductive organ development (ovary and egg formation), and larval body melanin pigmentation. This study provides the first evidence that the *BmAWH* gene exhibits pleiotropic roles across multiple developmental processes in *B. mori*.

## 1. Introduction

Homeobox genes contain a highly conserved 180-base pair sequence known as the homeobox, and this sequence encodes a 60-amino acid homeodomain, a motif essential for binding DNA and regulating gene expression during development [1]. Homeodomain (HD) is a multifunctional family of transcription factors that act as master regulators in various developmental processes of eukaryotes, including *Drosophila melanogaster* [2,3]. Proteins belonging to the LIM class are characterized by an additional conserved region located N-terminal to the homeodomain (LIM domain) [4]. LIM homeodomain (LIM-HD) proteins are a subclass of transcription factors characterized by the presence of two LIM zinc finger motifs and a homeodomain [5,6]. These dual domains enable LIM-HD proteins to function as both DNA-binding regulators and scaffolds for protein–protein interactions, thereby allowing them to coordinate complex developmental processes [7,8]. Researchers have identified several genes encoded for the LIM-HD protein including *Apterous* (*ap*), *Arrowhead* (*AWH*), *Xlim-3*, *lim1-6*, *Gsh-4*, *isl 1*, *lhx1-9*, and *lmx1b* from various organisms such as African clawed frogs, rats, and *D. melanogaster* [9,10,11,12,13,14,15]. Moreover, it was reported that LIM homeobox (lhx) genes were expressed in complementary patterns along the cephalic ganglia and digestive system of the adult planarian flatworms *Schmidtea mediterranea* [16]. The function of multiple LIM-HD genes and their co-regulators were also reported to be expressed in a sexually dimorphic pattern in developing mouse gonads [17]. While gene manipulation techniques have been implemented in various organisms in order to explore the function of LIM-HD encoding genes, no studies, however, have systematically assessed the pleiotropic (a single gene influencing two or more traits) effects caused by the overexpression of LIM-HD genes during insect development in silkworm *Bombyx mori*.

Within the LIM classes, the *Arrowhead* (*AWH*) is included in the Lmx subgroup, which consists of the LIM-HD transcription factor 1 alpha (*Lmx1a*) and LIM-HD transcription factor 1 beta (*Lmx1b*) [18]. Previous studies reported that the *B. mori Arrowhead* gene (*BmAWH*) acts as a key transcription factor that activates three silk fibroin genes (*fib-H*, *fib-L*, and *fh-x*) in silkworms [19,20]. The silk gland of *B. mori* is a highly specialized secretory organ responsible for the synthesis of fibroin and sericin proteins, which form the structural basis of the cocoon. Its development and function are tightly regulated by transcription factors that control tissue-specific gene expression. In *D. melanogaster*, the *AWH* gene has been shown to play a critical role in salivary gland morphogenesis and the activation of gland-specific secretory genes [21,22]. Moreover, the study of *AWH* overexpression affecting various tissues in silkworm *B. mori* has not yet been explored. The full extent of their functional diversity and the consequences of the *AWH* overexpression, particularly in holometabolous insects like *B. mori*, deserve further exploration. The functional role of *BmAWH* can be inferred by comparing its expression profile and associated phenotypes with homologous genes described in species of other insect orders.

Among Lepidopteran insects, the silkworm (*Bombyx mori*) is one of the most suitable model systems for gene manipulation such as CRISPR/Cas9 and piggyBac-based transformation [23,24]. Owing to the function of *BmAWH* as a key developmental regulator, its overexpression could alter the spatial and temporal developmental programs leading to diverse morphological and physiological outcomes. The objective of this study is to investigate the functional role of a *BmAWH* gene in *B. mori* (Dazao) development by analyzing the phenotypic consequences of its overexpression. We presented the emergence of multiple distinct phenotypes affecting silk gland development and cocoon traits, larval body morphological patterning (melanin pigmentation), and reproductive organ development at different developmental stages. This study provides the first evidence showing that the *AWH* gene exhibits pleiotropic roles across multiple developmental processes in *B. mori*. Investigating the pleiotropic role of the *AWH* gene is crucial, as it connects silk production, reproduction, and pigmentation, because these traits are the vital traits for both biology and sericulture fields. Uncovering these links advances our understanding of insect developmental genetics while providing practical avenues to enhance silk yield and offering broader insights into gene regulation and evolution.

## 2. Materials and Methods

### 2.1. Silkworm Rearing

In this study, silkworm *Bombyx mori* Dazao were obtained from the National Silkworm Genetic Resources Bank (Southwest University, Chongqing, China), and they were used for the *BmAWH* transgenic injection [24]. Dazao eggs were incubated under full light at 25 °C, and larvae were reared at a constant temperature of 25 °C, with relative humidity of 75–85%. The larvae were fed with fresh mulberry leaves at regular intervals daily, and pupae were kept at 25 °C until adult moth eclosion. Tissue samples, including silk gland (Sg), head (He), epidermis (Ep), hemolymph (Hem), intestine (In), fat body (Fb), testis (Te), and ovary (Ov), were collected from Dazao at larval and pupal stages for *BmAWH* expression analysis. All larvae and pupae were anesthetized and dissected under a stereomicroscope (SZX7, Olympus Corporation, Shinjuku, Japan) using fine forceps and scissors. All tissues and embryos were pooled in RNase-free tubes with ice-cold PBS. Each complete organ (e.g., ovary, testis, and silk gland) was placed individually into a separate RNase-free 1.5 mL tube to represent one biological replicate. Hemolymph was collected by puncturing a proleg and mixing immediately with anticoagulant buffer (4 μL of 0.2 M thiourea powder) and hemolymph samples were centrifuged at 3000× *g* and 4 °C for 30 min. The supernatant was transferred into new 1.5 mL EP tubes. All samples were stored at −80 °C until RNA extraction.

### 2.2. Construction of PiggyBac Transgenic Vectors and Generation of Transgenic Silkworms

The cDNA sequence of *BmAWH* was isolated from *B. mori* strain Dazao and used to amplify the PCR product. The following PCR profile was used to amplify target sequences: 98 °C for 2 min; 35 cycles of 98 °C for 10 s, 55 °C for 15 s, and 68 °C for 15 s; and 68 °C for 5 min. Amplicons were gel purified using High Pure PCR Product Purification Kit from Roche, and the pure amplicon was cloned into the pMD19-T simple plasmid (Takara Bio, Kyoto, Japan). Confirmation of *BmAWH* sequence was obtained by sequencing (BGI, Shenzhen, China) and subjected to the BLASTn query sequence on SilkBase website (https://silkbase.ab.a.u-tokyo.ac.jp/cgi-bin/index.cgi, accessed on 7 October 2023). The transgenic vector piggyBac[3xP3-EGFP, IE1-*BmAWH*-SV40] was successfully constructed by Beijing Genomics Institute (BGI) (Beijing, China). All primers used for PCR and cloning are listed in Table S1. For generation of transgenic silkworms, newly laid nondiapause Dazao silkworm eggs, within 2 h, were used for the transgenic vector injection using 1:1 ratio mixture of the donor plasmid piggyBac[3xP3-EGFP, IE1-*BmAWH*-SV40] and the helper plasmid pHA3PIG. The injected eggs were incubated at a constant temperature of 25 °C and with 90% humidity until hatching. The hatched larvae were reared under a constant temperature of 25 °C with 75–80% humidity. The G0 generation individuals were crossbred with wild-type (WT) Dazao to obtain the G1 generation eggs. The positive individuals were screened by observing the presence of fluorescence in the eyes under a stereo fluorescence microscope (Leica, Wetzlar, Germany) indicating the successfully expressed EGFP. The DNA of positive transgenic lines were extracted from head of larvae using the DNAiso reagent (TaKaRa, Kyoto, Japan), following the manufacturer’s instructions, and subjected to PCR, and the primers used are listed in the Table S1. The insertion site of *BmAWH* was confirmed by sequencing.

### 2.3. RNA Extraction and qRT-PCR

Total RNA samples were isolated from the different tissues of silkworm Dazao at different developmental stages using an E.Z.N.A.^®^ Total RNA Kit I (Omega Bio-Tek, Guang Zhou, China) according to the manufacturer’s instructions. All total RNA extracted were stored in −80 °C until used. The first-strand cDNA was synthesized using PrimeScript™ RT Master Mix (Perfect Real Time) (TaKaRa). The real-time quantitative PCR (qRT-PCR) mixture was prepared using SYBR^®^ Green Premix Pro Taq HS qPCR Kit (AG, Changsha, China), following the manufacturer’s protocol, and the amplicon analysis was performed using an qTOWER3G system (Analytikjena, Jena, Germany). *RP49* (*KWMTBOMO02081*) was used as the internal control genes and the relative expression levels were calculated using the 2^−ΔΔCt^ method. All primers used are listed in Table S1, and the gene expression profile results were obtained with three biological replicates and three technical replicates.

### 2.4. Protein Extraction and SDS-PAGE Analysis

The whole ovaries from virgin female moths of both the wild-type (WT) and the transgenic line (*BmAWH*-OE-F) were dissected. Ovaries were carefully dissected under a dissecting microscope (SZX7, Olympus Corporation, Shinjuku, Japan), using fine forceps in ice-cold phosphate-buffered saline (PBS, pH 7.4). Each ovary was placed individually into a separate 1.5 mL Eppendorf tube, such that every tube contained the complete ovary from a single moth. A total of 500 µL of RIPA protein lysis buffer (Solarbio, Beijing, China) was added into the tube, and the ovaries were crushed using metal beads beating method and homogenizer (60 Hz, 180 s). The samples were centrifuged at 12,000 rpm, 4 °C for 20 min, and the supernatant was taken and transferred into the new, clean 1.5 mL EP tube. The extracted protein samples were kept in −20 °C until used. The total concentration of protein was detected using BCA assay kit following the manufacturer’s instructions (Beyotime Biotechnology, Shanghai, China). A total of 50 μg of protein concentration per sample was resolved by SDS-PAGE on 8% and 3–12% (*w*/*v*) polyacrylamide gradient gels for detection of proteins related to ovary development processes: Vitellogenin large subunit (BmVn-H), Vitellogenin small subunit (BmVn-L), egg specific protein (ESP), 30K protein (30K), respectively, according to the method of Laemmli [25]. Appropriate amounts of Coomassie Brilliant Blue rapid staining solution were used to stain the gel. SDS-PAGE gels were photographed by Bio-Rad GS-900 Calibrated Densitometer (Bio-Rad, Hercules, CA, USA).

### 2.5. Measurement of Juvenile Hormone (JH) Titer Level

Hemolymph samples were collected from the wild-type Dazao female (DZ-F) silkworms and overexpression transgenic lines (*BmAWH*-OE-F) during the fifth instar (5L) larval stage (day 1, 2, 3, 5, and 7), following the protocol mentioned previously. JH concentrations in silkworm hemolymph were quantified using the JH ELISA kit (Meimian, Yancheng, China), following the manufacturer’s instructions. A total of 10 µL of hemolymph samples were used for every reaction. The study involved selecting the same batch of silkworms with the same body weight. Finally, the OD value of each sample was measured at a wavelength of 450 nm using a BioTek Synergy H1 microplate reader. The standard solution (concentrations: 0, 7.5, 15, 30, 60, and 120 ng/mL) supplied with the kit was used for standard curve preparation, and sample concentrations were calculated based on the kit protocol provided by the manufacturer. All samples were analyzed with three biological replicates, and each measurement was performed in triplicate, as technical replicates.

### 2.6. Phylogenetic Analysis

Detailed *Bombyx mori Arrowhead* gene *(BmAWH*) information and whole sequence were retrieved from SilkMeta website (http://silkmeta.org.cn, accessed on 20 September 2022) [26]. National Center for Biotechnology Information (NCBI) (http://www.ncbi.nlm.nih.gov/, accessed on 21 September 2022) BLAST Service was utilized to perform a nucleotide–nucleotide (BLASTn) and protein–protein BLAST search (BLASTp) to identify homologous proteins of *BmAWH* in various insect orders and species, and the accession numbers are listed in the Table S2. The conserved domain search was obtained using SMART (http://smart.embl-heidelberg.de/, accessed on 1 August 2024), and protein structure was predicted on Protein Data Bank website (https://www.rcsb.org/, accessed on 1 January 2025). The genomic structure, predicted domain, and protein structure were conducted. Amino acid sequence alignments were conducted using the ClustalW program, and the phylogenetic tree was constructed using the neighbor-joining method (NJ) and assessed through the bootstrap method with 1000 replicates, using the Molecular Evolutionary Genetics Analysis 11 (MEGA 11) software.

### 2.7. Statistical Analysis

All values of the differences between groups (mean ± SD) were analyzed with Student’s *t*-tests, and differences among multiple groups were analyzed using two-way ANOVA, followed by Bonferroni post hoc test for pairwise comparisons, using GraphPad Prism 8 (GraphPad Software). A value of *p* < 0.05 was considered to be significant; ns, nonsignificant; * *p* < 0.05; ** *p* < 0.01; *** *p* < 0.001; and **** *p* < 0.0001.

## 3. Results

### 3.1. Gene Cloning, Molecular Characterization, and Expression Level of BmAWH

**Figure 1:**
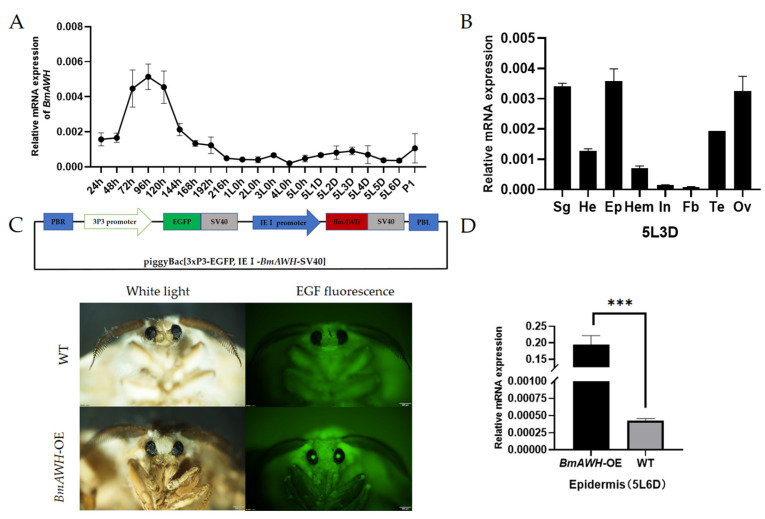
Temporal expression levels of *BmAWH* in silkworm Dazao and construction of recombinant plasmid and transgenic silkworms. (**A**) The expression level of *BmAWH* from whole body of silkworm started from after oviposition (newly laid eggs) (24 h, 48 h, 72 h, 96 h, 120 h, 144 h, 168 h, 192 h, and 216 h), first larval instar stage (1L0h), second larval instar stage (2L0h), third larval instar (3L0h), fourth larval instar (4L0h), fifth larval instar (5L0h day 6), and day 1 pupal stage (P1). The expression level is highest at 96 h. (**B**) Expression level of *BmAWH* in different tissues at fifth instar on day 3 (5L3D); silk gland (Sg), head (He), epidermis (Ep), hemolymph (Hem), intestines (In), fat body (Fb), testis (Te), and ovary (Ov). The highest expression level of *BmAWH* was observed in epidermis (Ep), second highest was silk gland (Sg) and ovary (Ov) at fifth instar on day 3 (5L3D) of Dazao. (**C**) The schematic illustration of the piggyBac[3xP3-EGFP, IE1-*BmAWH*-SV40] vector and the isolation of successfully overexpressed transgenic line (G1) by detection of EGFP fluorescence in the eyes of moths. (**D**) Expression level of *BmAWH* in epidermis of transgenic line *BmAWH*-OE at fifth instar on day 6 (5L6D) was significantly higher compared to wild-type Dazao (WT). *** indicate that the *p*-values of Student’s *t*-tests are less than 0.001 and error bars represent mean ± SE.

To identify the molecular structure and to predict the functions of *Bombyx mori Arrowhead* genes (*BmAWH*), the CDS region of *BmAWH*, which is located at chromosome 1, was successfully cloned in a pMD19-T vector. The clone was verified by sequencing, and all predicted function and molecular characterization information of the gene was referred to the Silkmeta and SilkDB 3.0 websites (Figure S1A). Figure S1 represents the molecular structure, predicted domain, and protein structure of BmAWH. BmAWH protein domains were composed of two LIM domains at 6aa-59aa and one Hox domain at 67aa-121aa (Figure S1A). Figure S1B represents the predicted protein structure shown by the crystal structure of Isl1 LIM domains, with the Ldb1 LIM-interaction domain composed of the insulin gene enhancer protein. The expression level of *BmAWH* from the whole body of the silkworm was evaluated, starting after oviposition at 24 h, 48 h, 72 h, 96 h, 120 h, 144 h, 168 h, 192 h, and 216 h, the first larval instar stage (1L0h), second larval instar stage (2L0h), third larval instar (3L0h), fourth larval instar (4L0h), fifth larval instar (5L0h day 6), and day 1 pupal stage (P1). It was observed that the expression levels of the *BmAWH* gene were highest at 96 h of the Dazao embryonic stage (Figure 1A). Moreover, the gene expression level of *BmAWH* in different tissues of Dazao was evaluated at the fifth instar on day 3 (5L3D) (Figure 1B). The tissues evaluated are the silk gland (Sg), head (He), epidermis (Ep), hemolymph (Hem), intestines (In), fat body (Fb), testis (Te), and ovary (Ov). It was observed that the *BmAWH* gene was expressed in all tissues and highly expressed in Ep, Sg, and Ov (Figure 1B). The expression level of *BmAWH* was relatively lower in He, Tes, Hem, and In but lowest in Fb. These results suggest that the *BmAWH* gene may be a key regulatory factor to maintain the development of the silk gland, epidermis, and ovary of Dazao.

### 3.2. Construction of BmAWH Transgenic Vector and Transgenic Silkworms

The ORF of *BmAWH* was successfully inserted into the recombinant vector piggyBac[3xP3-EGFP, IE1-*BmAWH*-SV40] and the vector constructed, as shown in the schematic illustration (Figure 1C). Hatched G0 larvae were reared to adult moths and crossed with the wild-type moths. The successfully overexpressed transgenic line (G1) was screened by the detection of EGFP fluorescence in the eyes of moths (Figure 1C). The positive individuals were raised until the seventh generation and maintained under a controlled temperature. The DNA of positive transgenic lines were extracted, and the insertion site of *BmAWH* was detected by sequencing the PCR product. The insertion site was located at chromosome 8, 830 bp between 26, 10718 and 26, 11546. To confirm the overexpression of transgene lines, the expression level of *BmAWH* was determined using a qRT-PCR. The expression level of *BmAWH* in the epidermis of transgenic line *BmAWH*-OE at the fifth instar on day 6 (5L6D) was significantly higher compared to wild-type Dazao (WT) (Figure 1D).

### 3.3. BmAWH Overexpression Reduced the Size of the Silk Gland and Reduced Cocoon Traits

**Figure 2:**
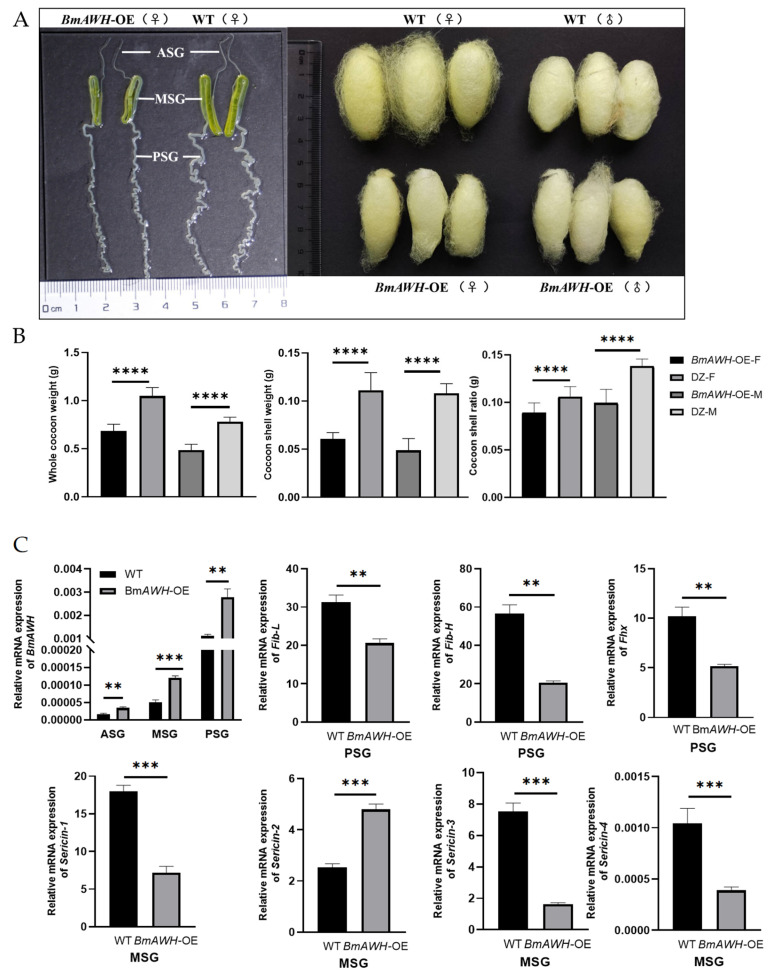
Effects of overexpression of *BmAWH* gene on silk gland, silk yield, and silk synthesis genes. (**A**) Silk gland morphology (anterior silk gland (ASG), middle silk gland (MSG), and posterior silk gland (PSG)) observation at 5th instar day 7 (5L7D) of the female wild-type Dazao (WT) and *BmAWH*-OE transgenic lines and cocoon morphology observation. (**B**) Differences in whole cocoon weight (WCW), cocoon shell weight (CSV), and cocoon–shell ratio (CSR) between wild-type Dazao (WT) and the transgenic overexpression line of wild-type Dazao males (DZ-M) and females (DZ-F) and *BmAWH*-OE transgenic line males (*BmAWH*-OE-M) and *BmAWH*-OE transgenic line females (*BmAWH*-OE-F). (**C**) Expression level of *BmAWH* gene in ASG, MSG, and PSG of wild-type Dazao (WT) and *BmAWH*-OE at 5L7D, and fibroin-related genes, fibroin-light-chain (*Fib-L*), fibroin-heavy-chain (*Fib-H*), and fibrohexamerin (*Fhx*) from PSG and sericin-related genes (*Sericin-1*, *Sericin-2*, *Sericin-3*, and *Sericin-4*) from MSG at 5L7D. **, ***, and **** indicate that the *p*-values of Student’s *t*-tests are less than 0.01, 0.001, and 0.0001, respectively. Error bars represent mean ± SE.

Morphological observation of the silk gland size and cocoon traits, including cocoon size and weight, were made in the *BmAWH*-OE transgenic lines. It was observed that the size of the silk gland of *BmAWH*-OE transgenic line femaled at the fifth instar on day 7 (5L7D) had become smaller, especially the posterior silk gland (PSG), which had become shorter and thinner (Figure 2A); however, quantitative measurements were not performed. A smaller size of cocoon was produced by *BmAWH*-OE transgenic lines, where both the female and male were also significantly smaller (Figure 2A). Consequently, the smaller size of the silk gland adversely affected cocoon traits such as whole cocoon weight (WCW), cocoon shell weight (CSW) and the cocoon–shell ratio (CSR) (Figure 2A). The quantitative data of cocoon traits (WCW, CSW, and CSR) of 20 male and female transgenic silkworms and wild-type Dazao (WT) were investigated, and data is listed in Tables S3 and S4 (n = 20). Compared to the WT, the WCW of female *BmAWH*-OE transgenic lines decreased by 37% and that of males by 30%. The CSW of female *BmAWH*-OE transgenic lines decreased by 5% and that of males by 6%. The CSR of female *BmAWH*-OE transgenic lines decreased by 2% and that of males decreased by 4% (Figure 2B). To further investigate the underlying causes of silk gland shrinkage and the reduction in cocoon traits in *BmAWH*-OE transgenic lines, the total RNA was extracted from the whole silk gland of the wild-type and *BmAWH*-OE transgenic lines. Total RNA was extracted from the anterior silk gland (ASG), middle silk gland (MSG), and posterior silk gland (PSG) at 5L7D for evaluation of the *BmAWH* gene level. The expression level of the *BmAWH* fibroin-related genes, fibroin-light-chain (*Fib-L*), fibroin-heavy-chain (*Fib-H*), and fibrohexamerin (*Fhx*) and sericin-related genes (*Sericin-1*, *Sericin-2*, *Sericin-3*, and *Sericin-4*) were evaluated using a qRT-PCR. The result showed that the *BmAWH* gene was highly expressed in *BmAWH*-OE transgenic lines compared to WT at PSG (*p*-value = 0.0062), ASG (*p*-value = 0.0023), and MSG (*p*-value = 0.0002) (Figure 2C). Interestingly, the expression levels of three silk protein genes were significantly downregulated in the PSG of *BmAWH*-OE transgenic lines: *Fib-L* (*p*-value = 0.0027), *Fib-H* (*p*-value = 0.0002), and *Fhx* (*p*-value = 0.0019) (Figure 2C). In addition, the expression of three sericin genes, *Sericin-1*, *Sericin-3*, and *Sericin-4*, in MSG was significantly downregulated (*p*-values < 0.0001) in *BmAWH*-OE transgenic lines, but the expression level of *Sericin-2* was upregulated (*p*-value < 0.0001) in *BmAWH*-OE transgenic lines.

### 3.4. BmAWH Overexpression Leads to Female Reproductive Defects in Adult Moths

**Figure 3:**
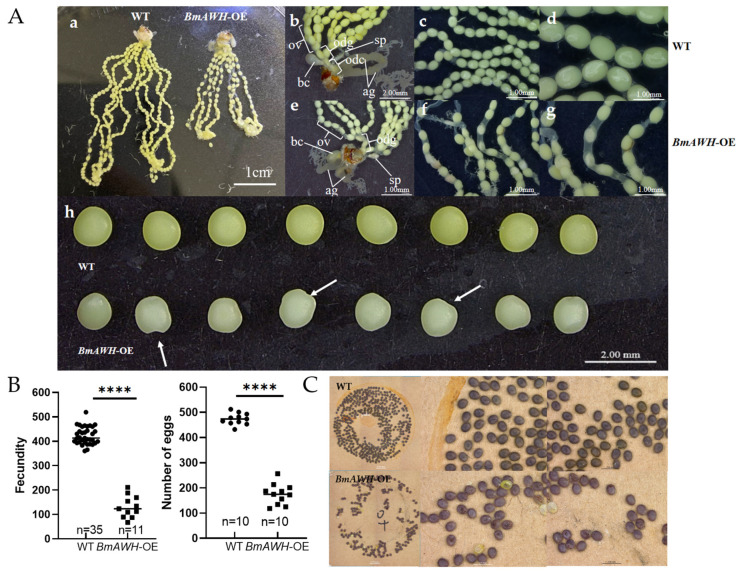
Effects of *BmAWH* overexpression on female reproductive organ development and oviposition. (**A**) Morphological observation of reproductive organ sizes and structures. (**a**) Left: wild-type Dazao (WT); right: transgenic overexpression line (*BmAWH*-OE); (**b**–**d**) internal reproductive organs of wild-type DZ female moths, close-up images of ovarian tubes in WT; and (**e**–**g**) close-up images of ovarian tubes in *BmAWH*-OE transgenic lines. Ovarian tube (Ov); spermatheca (sp); common oviduct (odc); accessory gland (ag); lateral oviduct (odg); bursa copulatrix (bc); and (**h**) the white arrows point the morphology and color of eggs. (**B**) Fecundity rate and total number of eggs inside the moths. (**C**) Diapause eggs produced by both DZ (WT) and transgenic overexpression lines (*BmAWH*-OE) moths. **** indicate that the *p*-values of Student’s *t*-tests are less than 0.0001, respectively. Error bars represent mean ± SE.

To examine whether *BmAWH* overexpression affects reproductive development in adult moths, we analyzed the morphology and reproductive capacity of *BmAWH*-OE transgenic lines. A total of 10 virgin moths were dissected, and the whole ovaries and reproductive organs were observed in wild-type (WT) and *BmAWH*-OE transgenic lines. It was found that the size of the whole ovaries of *BmAWH*-OE transgenic lines was significantly smaller than the wild-type’s (WT) (Figure 3A(a)). Observation of the female moth’s internal reproductive organs revealed that the ovarian tube (Ov), spermatheca (sp), common oviduct (odc), accessory gland (ag), lateral oviduct (odg), and bursa copulatrix (bc) were intact and showed no obvious abnormalities in *BmAWH*-OE (Figure 3A(b–g)). Moreover, the ovarian tubes of the female moths of the *BmAWH*-OE were significantly shortened, the number of eggs was significantly reduced, and the eggs were not arranged tightly (Figure 3A(f,g)). However, some of the *BmAWH*-OE eggs had abnormal shapes, which were concave from the edge of the egg to the inside, and some of the eggs did not consist of yolk protein in the entire egg, and the eggshell and internal yolk were not connected. The color of eggs of the WT group was yellow, but the eggs of *BmAWH*-OE transgenic lines were obviously lighter (pale yellow) (Figure 3A(h)). Due to reproductive impairment, the number of eggs laid (fecundity) by the *BmAWH*-OE moths also significantly decreased by 68.99% (Figure 3B). It was observed that *BmAWH* overexpression may have interfered with the ovarian development and oogenesis of female adult moths, leading to the production of abnormal egg morphology. These physiological defects were associated with a decreased fecundity rate and reduced the number of viable eggs produced. Together, these outcomes indicate that *BmAWH* plays a crucial role in regulating reproductive competence in female silkworm (ovary) adult moths. The eggs produced by both WT and *BmAWH*-OE transgenic lines have entered the diapause state after three days of oviposition, observed by the dark brown color, as shown in (Figure 3C). It is concluded that the *BmAWH* overexpression not only led to female reproductive defects but also caused lethality to the next generation (embryonic stage).

**Figure 4:**
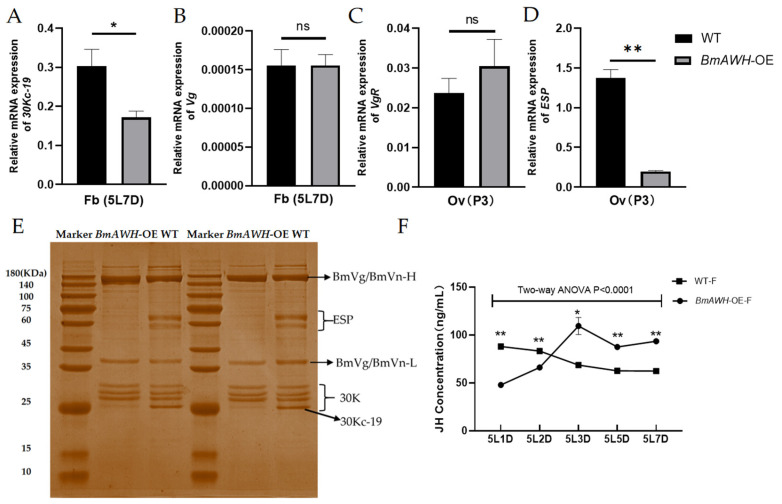
Comparison of the expression level of major genes related to egg and ovarian development and juvenile hormone (JH) titer between *BmAWH*-OE lines and wild-type silkworms. (**A**) Expression level of *30Kc-19* at fifth instar on day 7 (5L7D) in fat body (Fb), (**B**) *Vg* at fifth instar on day 7 (5L7D) in Fb, (**C**) *VgR* at day 3 pupal stage (P3) in ovary (Ov), and (**D**) *ESP* at day 3 pupal stage (P3) in Ov. (**E**) SDS-PAGE: BmVg protein (35–45 KDa), Vitellogenin large subunit (BmVn-H), Vitellogenin small subunit (BmVn-L), egg-specific protein (ESP) (72 KDa and 64 KDa), and 30K protein (30K) (25 KDa). (**F**) Juvenile hormone (JH) concentration level in the hemolymph of female *BmAWH*-OE transgenic lines (*BmAWH*-OE-F) compared to wild-type (WT) at fifth instar larval stage on day 1 (5L1D), day 2 (5L2D), day 3 (5L3D), day 5 (5L5D), and day 7 (5L7D) * and ** indicate that the *p*-values of Student’s *t*-tests and two-way ANOVA are less than 0.05 and 0.01 and ns indicates nonsignificant respectively. Error bars represent mean ± SE.

To further investigate the cause of the smaller size of ovaries and abnormal shape of eggs produced by *BmAWH*-OE transgenic lines, the fat body of female larvae at the fifth instar on day 7 (5L7D) and the ovaries of the 3-day-old pupal (P3) stage were extracted to verify the expression levels of the related genes by fluorescence qRT-PCR. All experiments were performed with nine biological replications, and each measurement was conducted in triplicate as technical replications. The quantitative results showed the expression level of *30Kc-19* (Figure 4A) and *Vg* (Figure 4B) at 5L7D and *ESP* (Figure 4C) and *VgR* (Figure 4D) at P3. The expression level of *30Kc-19* at 5L7D (*p* = 0.0282) and *VgR* at P3 were significantly downregulated (*p* = 0.0106) in *BmAWH*-OE transgenic lines, but there is no significant change in the expression of *Vg* at 5L7D and *ESP* at P3. It was reported that the 30K protein is mainly synthesized in the fat body at the end of the fifth instar, and the female *Bombyx mori* accumulates a large amount of egg proteins, mainly Vg, 30K, and egg-specific protein (ESP), during egg formation to provide the nutrition for embryo development [27]. To further investigate the cause of reproductive impairment due to *BmAWH* overexpression, total protein was extracted from the ovaries (virgin moths) of WT and *BmAWH*-OE transgenic lines, and the presence of proteins was detected. SDS-PAGE results obtained showed the protein expression levels of 30Kc-19 (25 KDa) and ESP (72 KDa and 64 KDa) were downregulated in the eggs of *BmAWH*-OE transgenic lines compared to the WT (Figure 4E). While there are no changes for the expression levels of the BmVg protein (35–45 KDa), which consists of the Vitellogenin large subunit (BmVn-H) and Vitellogenin small subunit (BmVn-L) in *BmAWH*-OE transgenic lines (Figure 4E). Strikingly, the juvenile hormone (JH) levels in hemolymph of *BmAWH*-OE transgenic lines at the fifth instar (5L) larval stage on day 1 (5L1D) and day 2 (5L2D) were significantly reduced compared to the WT group, but the JH levels were dramatically increased at day 3 (5L3D), day 5 (5L5D), and day 7 (5L7D) (Figure 4F). It was expected that the elevation of JH during the fifth instar larval stage was associated with the changes in the expression levels of *30Kc-19* and *VgR*, affecting the synthesis of ESP and 30K proteins.

### 3.5. BmAWH Overexpression Resulted in Loss of Larval Melanin Pigmentation

**Figure 5:**
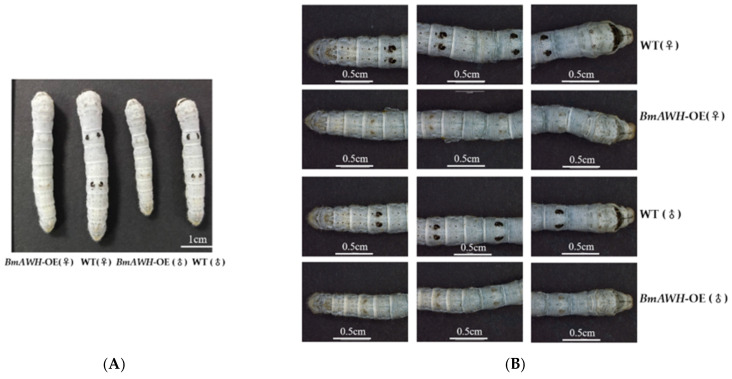

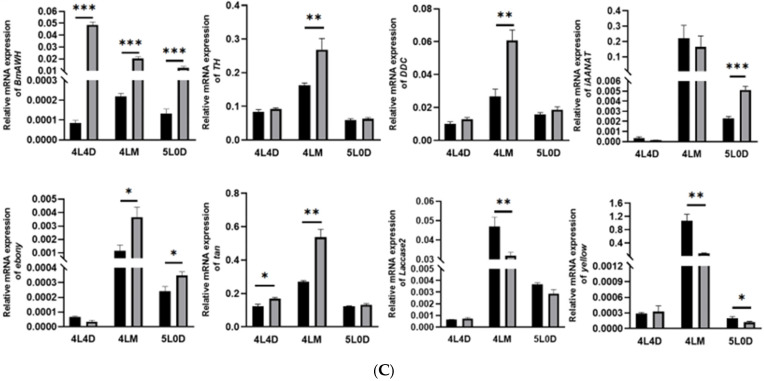
Effects of *BmAWH* overexpression on larval body coloration and the expression of melanin synthesis-related genes. (**A**) Morphology of larval body of *BmAWH*-OE transgenic lines for both males and females. (**B**) Observation of the disappearance of characteristic spot patterns in *BmAWH*-OE male and female lines. (**C**) The expression of *BmAWH* and melanin synthesis pathway-related genes (*TH*, *DDC*, *ebony*, *tan*, *iAANAT*, *laccase2*, and *yellow*) was evaluated in epidermal tissues at fourth instar molting time (4L4D), fourth instar molting time (4LM), after the ecdysis, and fifth instar at 0 h (5L0D). *, **, and *** indicate that the *p*-values of Student’s *t*-tests are less than 0.05, 0.01, and 0.001 respectively. Error bars represent mean ± SE.

We analyzed the morphology of *BmAWH-OE* transgenic lines to determine whether *BmAWH* overexpression alters body patterns and the melanin pigmentation of silkworm larvae. It was observed that the overexpression of *BmAWH* affected the melanin pigmentation, causing visible changes on the surface of the larval body including the markings and pigmentation during the whole larval stage in both female and male larvae (Figure 5A). All *BmAWH*-OE transgenic larvae exhibited a significant reduction in epidermal melanin pigmentation, with the black spots on the body surface disappearing, including eye spots on the second thoracic segment, crescent patterns on the second abdominal segment, star-shaped spots on the fifth abdominal segment, and other small dot-shaped spots, but the light yellow marks remained visible (Figure 5B). The *BmAWH*-OE transgenic lines were raised for seven generations, and this phenotype was observed in every generation (about 400 larvae were raised per generation). Insects synthesize their own pigments, which form solid particles that become embedded in the cuticle of their body surface and the scales of their wings [28]. The expression level of genes related to the melanin metabolism pathways: *TH*, *DDC*, *iAANAT*, *ebony*, *tan*, *laccase2*, and *yellow*, were evaluated to verify if this phenotypic changes at 4L4D, 4LM, and 5L0D (Figure 5C). The epidermis area, which covered the range of the eye-spots shape, crescent patterns, and star-shaped spots of the WT and *BmAWH*-OE transgenic lines, were collected before the fourth instar molting time (4L4D), during the fourth instar molting time (4LM), and just after the ecdysis fifth instar at 0h (5L0D); the total RNA was extracted for the qRT-PCR. It was observed that the expression level of *BmAWH* was higher in *BmAWH*-OE transgenic lines at 4L4D, 4LM, and 5L0D compared to the WT, and it was significantly highest at 4L4D (Figure 5C), with a *p*-value < 0.0001. Interestingly, the expression levels of *TH*, *DDC*, *ebony*, and *tan* genes increased significantly, especially at the fourth molting time (4LM) in *BmAWH*-OE transgenic lines compared to WT (*TH* (*p* = 0.0201), *DDC* (*p* = 0.0058), *ebony* (*p* = 0.0142), and *tan* (*p* = 0.0118)). Meanwhile, the expression levels of *iAANAT* and *ebony* increased significantly at 5L0D (*iAANAT* (*p* = 0.0001) and *ebony* (*p* = 0.0273)), and the expression levels of *laccase2* and *yellow* genes were significantly downregulated at 4LM (*laccase2* (*p* = 0.0085) and *yellow* (*p* = 0.0003)) (Figure 5C). It is speculated that the overexpression of *BmAWH* leads to elevated expression levels of *BmAWH* prior to molting at 4L4D, thereby altering the expression of melanin pigment synthesis genes, particularly during the molting stage at 4LM.

### 3.6. Phylogenetic Analysis of BmAWH Across Different Insect Orders

To evaluate the diversity and evolutionary conservation of *Arrowhead* (*AWH*) genes within and across the orders, the *AWH* amino acid sequences from across 30 insect species, representing six major insect orders (Lepidoptera, Orthoptera, Blattodea, Cleoptera, Diptera, and Hymenoptera), were compared, and a phylogenetic tree was constructed. The neighbor-joining (NJ) method was implemented based on the aligned amino acid sequences, performed with the bootstrap method and 1000 replicates, using the Molecular Evolutionary Genetics Analysis 11 (MEGA 11) software. The phylogenetic tree constructed illustrates the evolution of *BmAWH* across intra- and inter-orders (Figure S2). *Bombyx mori Arrowhead* genes (*BmAWH*) fall within the Lepidotera order, and there are 16 species of the Lepidoptera order, which were aligned using ClustalW. The sequence homology analysis revealed 60–70% identity among species within the order Lepidoptera, and *BmAWH* was grouped together with *Melitaea cinxia* and *Danaus plexippus* (butterflies) with a 62% homology. In contrast, the sequence identity between Lepidoptera and species from Orthoptera or Blattodea was less than 70%, whereas homology between Lepidoptera and species from Diptera or Hymenoptera exceeded 70%. In a phylogenetic analysis, <50% is considered low homology, while 60–70% is considered moderate and >70% is considered high homology. There are moderately conserved regions across orders, highlighting the accumulation of differences that likely contribute to the diversification of traits and adaptation to the unique niches occupied by different orders. Thus, it is expected that the function of *AWH* could be varied in different species and insect orders. Such divergence may reflect the functional diversification of the gene in different lineages, potentially adapting to specific ecological or physiological requirements.

## 4. Discussion

In this present study, the *Bombyx mori Arrowhead* (*BmAWH*) gene was cloned and characterized, and the molecular characteristic was identified in order to understand its function in silkworm development. The expression level of *BmAWH* was evaluated, and the result showed that *BmAWH* exhibited high expression levels in the silk gland, ovary, and epidermis, suggesting its crucial role in the developmental processes of these tissues. Accordingly, we presented multiple phenotypic alterations observed in the silk gland, ovary, and epidermis upon *BmAWH* overexpression, suggesting the pleiotropic effects of the gene on silkworm development. *AWH* gene encodes the LIM homeodomain (LIM-HD), which is known as a transcription factor that plays a function in regulating the development of both abdominal histoblasts and salivary gland imaginal ring cells [22]. To better understand the functional role of *BmAWH*, we compared the gene expression pattern and associated phenotypes with homologous genes reported in species of other insect orders.

This present study revealed that the overexpression of *BmAWH* caused a smaller silk gland size and reduced the cocoon traits due to the downregulation of fibroin genes (*Fib-L*, *Fib-H* and *Fhx*) in the posterior silk gland (PSG) and sericin genes (*Sericin-1*, *Sericin-3*, and *Sericin-4*) in the middle silk gland (MSG) at fifth instar on day 7 at 5L7D. Interestingly, only the *Sericin-2* expression level was upregulated at 5L7D. In contrast to our findings, previous studies reported that the overexpression of *AWH* in transgenic silkworm *B. mori* induced ectopic expression of the three fibroin genes (*Fib-L*, *Fib-H*, and *Fhx*) at the fifth instar on day 2 (5L2D) in MSG instead of PSG after heat treatment [19]. In contrast to previous studies suggesting that *AWH* acts as a common activator of fibroin genes, our findings indicate a different regulatory outcome. This discrepancy may result from differences in promoter context, tissue specificity, developmental stage, or strain background, suggesting that *AWH* regulation of fibroin genes is context-dependent and may not act uniformly as an activator under all conditions. While the earlier study employed the *Fib-H* promoter to drive gene expression, our study utilized a standard overexpression vector, indicating that the observed gene activation can be reproduced using different expression systems. Moreover, the overexpression of *Fib-H* was only restricted to MSG cells, particularly in the middle part, suggesting the existence of additional regulators that reduce the occurrence of the inappropriate expression of *Fib-H* in other tissues [19]. A previous study reported that *Fib-L*, *Fib-H*, and *Fhx* were specifically expressed in PSG; thus, we evaluated the gene expression level in PSG rather than MSG [29]. The study proved that *AWH* binds to the specific cis-regulatory sequences in the transcriptional activator complex of the fibroin genes, silk gland factor-2 (SGF-2), via in vitro assay [19]. Hence, these results contradict the previous study, but there is no data that represents the cocoon traits in the previous study. In the present study, although *AWH* was likely still capable of binding SGF-2 and associating with fibroin gene regulatory regions, the overexpression of *AWH* may have altered the stoichiometric balance among SGF-2 components. Such an imbalance could impair complex assembly or stability, preventing optimal transcriptional activation. Alternatively, excessive *AWH* may have recruited transcriptional corepressors or triggered negative feedback regulation, thereby downregulating fibroin gene expression. These results suggest that the precise spatial and temporal control of *AWH* levels is critical for maintaining its activator function within SGF-2. In addition, the core silk fibers are coated by sericins, and the sericin genes (*Sericin-1*, *Sericin-2*, and *Sericin-3*) were reported to be expressed in the MSG [30]. The modification of fibroin gene expression in PSG by the overexpression of *AWH* can indirectly downregulate or alter sericin gene expressions, because the two sets of genes share the same cellular transcriptional resources and are part of mutually exclusive silk gland programs.

Previous studies reported the functions of *AWH* in fruit flies, *Drosophila melanogaster*, that act as an establishment of imaginal precursor cells, embryonic salivary gland development, and adult structure development [21,22]. The drosophila salivary gland secretes proteinaceous glue, used for pupal attachment, and was considered homologous to the silk gland of *B. mori* [31]. Loss-of-function or mutations of the *AWH* gene in drosophila have reduced the number of abdominal histoblasts and salivary gland cells before the proliferative stages of their development [22]. One of the drosophila salivary gland genes is *fkh*, which is required for salivary gland morphogenesis and for the expression of two salivary gland-specific structural proteins during late larval stages [32]. In both species, drosophila and *B. mori*, *fkh* acts as a key developmental regulator for the formation and functional identity of a large secretory gland (silk gland and salivary gland) but differ in transcription factors. In drosophila, the salivary gland formation is encoded by *extradenticle* (*exd*) and *homothorax* (*hth*) [33]. Based on the phylogenetic analysis, there is <70% homology of the *AWH* amino acid sequence of *Drosophila melanogaster* and *Drosophila elegans* (Diptera order) compared to the *BmAWH*. The cofactors and downstream gene networks controlled by *fkh* differ between the two species, reflecting the adaptation to different secretory products. Moreover, in both species, *AWH* acts downstream of *fkh*, which maintains gland identity and drives the expression of *AWH*. In short, the results of the overexpression of *BmAWH* represents the involvement of *BmAWH* as the transcription of silk protein synthesis pathway genes; however, its molecular mechanism in silk gland development is unknown. Further studies should be carried out to determine the function of *BmAWH* in the determination of cell numbers in silk glands and to evaluate cofactor regulation.

Earlier research demonstrated that the overexpression of *BmAWH* in silkworms modifies the silk gland genes, but there are no effects on any other tissues. Strikingly, in our study, we revealed that the overexpression of *BmAWH* not only affected the silk gland but also the reproductive organs of female silkworms. It was observed that *BmAWH*-OE transgenic lines produced smaller sizes of ovaries and abnormal shapes of eggs. The phenotype obtained was validated using qRT-PCR, and the result showed that the abnormality was caused by the downregulation of the egg synthesis genes, which are *30Kc-19* at the fifth instar on day 7 (5L7D) and *VgR* at the 3-day-old pupal (P3) in the fat body of *BmAWH*-OE transgenic lines. This result is parallel to the previous study conducted in silkworm *B. mori* about the function of *BmFhl2*, which encodes the FHL protein that consists of the four-and-a-half LIM domain [34]. The study revealed the function of BmFHL2 in egg formation via the 30K protein synthesis pathway in female *B. mori* [34]. Interestingly, gene manipulation of these two different genes yields a similar phenotype, suggesting that the domain itself plays a critical functional role. Both genes consist of the LIM domain and could bind to a similar regulator, as mentioned in the previous study that FHL2 is expressed in a variety of tissues, serving as a transcriptional regulator through its interaction with various transcription factors [35]. A recent study showed that the LIM-HD transcription factor 1 alpha (*HvLmx1a*) influences ovarian and testis morphology and development through lipid metabolism in *Henosepilachna vigintioctopunctata* (28-spotted lady beetle) using a gene-silencing technique (RNA-i) [36]. *H. vigintioctopunctata* is classified under the Coleoptera order, and knockdown of *HvLmx1a* not only caused abnormal ovary and testis development but also influenced molting, leading to death. Thus, it is expected that *BmAWH* also plays the same role as *HvLmx1a*, since *AWH* is also included in the Lmx subgroup, which consists of the LIM-HD transcription factor 1 alpha *Lmx1a* and *Lmx1b* [18]. *H. vigintioctopunctata* is classified in the Coleoptera order (beetles), and a phylogenetic tree analysis of the *AWH* amino acid sequence of Lepidoptera compared to Coleoptera species, such as *Tribolium madens*, *Tribolium castaneum*, *Leptinotarsa decemlineata*, *Zophobas morio*, and *Tenebrio molitor*, shows that there is relatively high homology (72%). Thus, we speculated that the *BmAWH* gene regulates the synthesis of 30K proteins, thereby affecting egg formation across insect orders. Further studies should be carried out to investigate the specific regulator that binds to the *AWH* in governing the egg synthesis pathways.

To further evaluate the function of *BmAWH* on regulating the development of silkworms in various tissues, morphology changes on the pigment of the silkworm larval body were observed. Surprisingly, there is a complete loss of larval body melanin pigmentation observed in the overexpression transgenic lines, suggesting disruptions in morphogenetic signaling and pigment biosynthesis pathways. The *AWH* gene is primarily involved in regulating the cell number during development, specifically within imaginal precursor tissues, rather than directly controlling cuticular or pigment functions. Although there are more than 220 genes encoded for cuticular proteins (CPs) in silkworm *B. mori* [37,38], *BmAWH* could be one of the crucial genes playing an indirect role related to the melanin synthesis pathways. Our results represent *BmAWH*-OE transgenic lines exhibiting the complete loss of melanin on the larval body, and the expression levels of melanin-related genes showed parallel results, as previous studies have reported [39]. Given that *AWH* encodes a LIM-homeodomain transcription factor, it has the potential to modulate a wide range of target genes, including pigment biosynthesis pathways. As mentioned before, the overexpression of *BmAWH* has induced the upregulation of *ebony*, *tan*, *TH*, and *DDC* at the fourth instar molting time (4LM), while downregulating the expression level of *iAANAT*, *laccase2*, and *yellow* at the 4LM. A previous study reported that the *mamo* gene belongs to the Broad complex, Tramtrack, and bric à brac/poxvirus zinc finger protein (BTB-ZF) family as a regulator of pigmentation in the silkworm *B. mori* [39]. Since we found out that *BmAWH* is expressed highly in the epidermis, and *BmAWH* encodes for protein that is composed of two LIM zinc finger motifs and a homeodomain, this gene could play similar role. Further studies are needed to identify the transcription factors that may participate in this pathway.

To the best of our knowledge, this is the first study reporting the overexpression of *BmAWH* in *B. mori* that leads to a broad range of developmental abnormalities and suggesting a pleiotropic role for this gene across different developmental stages. Pleiotropy was initially defined as the phenomenon whereby a single gene independently affects two or more phenotypes (traits) [40]. The observed phenotypes include underdeveloped silk glands, reduction in cocoon traits, abnormal female reproductive organs, and complete loss of larval body melanin pigmentation. A diverse group of LIM domain-containing proteins has been reported, exhibiting a wide range of functions such as gene regulation, cell fate determination, tumor development, and cytoskeleton organization [41]. Thus, we suggest, in addition to overexpression, future work could incorporate chromatin immunoprecipitation followed by sequencing (ChIP-seq) to define the DNA-binding landscape of *BmAWH*. Integration of ChIP-seq with RNA-seq datasets would allow discrimination between direct and indirect regulatory effects, thereby establishing a more comprehensive regulatory network model. Hence, these findings not only advance our understanding of silkworm biology but also have implications for genetic studies in other Lepidopteran insects and broader insect developmental research.

## 5. Conclusions

This study provides novel insights into the pleiotropic roles of the LIM-homeodomain gene *BmAWH* in *Bombyx mori*. Through piggyBac-mediated overexpression, we demonstrated that *BmAWH* influences multiple developmental pathways, including silk gland and cocoon development, reproductive organ formation, and morphological patterning across various life stages. These findings not only deepen our understanding of LIM-HD gene functions in insect development but also identify *BmAWH* as a key regulatory factor orchestrating multiple developmental processes in *B. mori*.

## Data Availability

Most of the analytical data are provided in the article. More original datasets used and analyzed during the current study are available from the corresponding author upon reasonable request.

## Supplementary Materials

The following supporting information can be downloaded at: https://www.mdpi.com/article/10.3390/biology14091248/s1, Figure S1. Predicted domain and protein structure of *BmAWH.* Figure S2. A phylogenetic tree was constructed using *Arrowhead* (*AWH*) amino acid sequence from across 6 different orders of insects which are Lepidoptera, Orthoptera, Blattodea, Cleoptera, Diptera, and Hymenoptera. Table S1. Primers used for PCR and qRT-PCR. Table S2. List of different insect species used for multiple sequence alignments and phylogenetic tree construction. Table S3. The quantitative data of cocoon traits of wild-type Dazao (WT) and *BmAWH*-OE transgenic lines (female). Table S4. The quantitative data of cocoon traits of wild-type wild-type Dazao (WT) and *BmAWH*-OE transgenic lines (male).
