# Supplementary material for: Overexpression of LIM Homeodomain Gene Arrowhead Induces Pleiotropic Developmental Alterations in the Silkworm, Bombyx mori
Source: Biology (Basel). 2025 Sep 11;14(9):1248. doi: 10.3390/biology14091248 (PMC12467131; doi:10.3390/biology14091248)
Supplement: Supplementary file 1 [file biology-14-01248-s001.zip › biology-3841410-supplementary.pdf]

## Supplementary Materials

Figures S1–S2

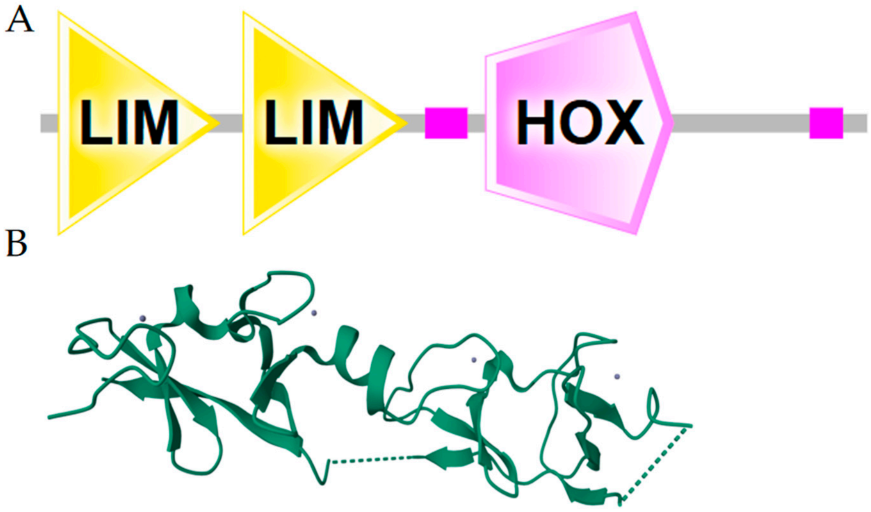


**Figure S1.** Predicted domain and protein structure of *BmAWH*. (**A**) Domains predicted by *BmAWH* gene in SMART showing *BmAWH* consist of LIM at 6aa-59aa and Hox domains at 67aa-121aa. (**B**) Protein predicted structure showed the crystal structure of Isl1 LIM domains with Ldb1 LIM-interaction domain composed of insulin gene enhancer protein.

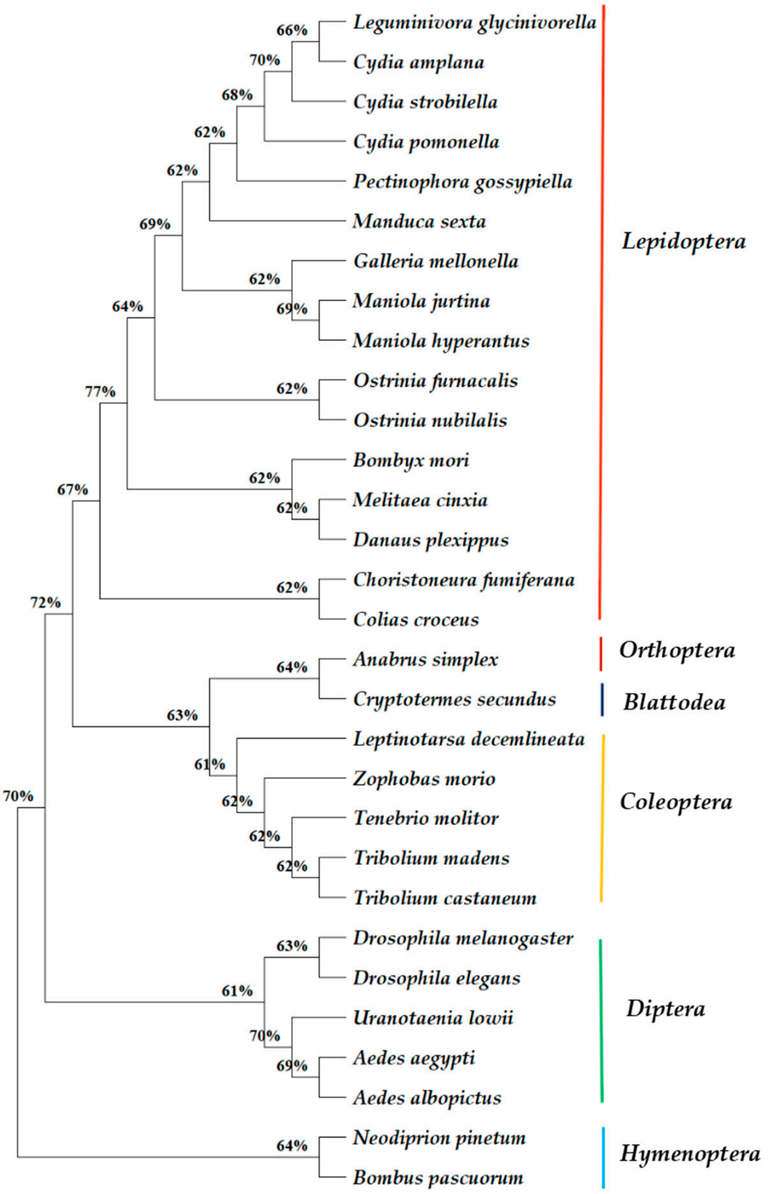


**Figure S2**. A phylogenetic tree was constructed using *Arrowhead* (*AWH*) amino acid sequence from across 6 different orders of insects which are Lepidoptera, Orthoptera, Blattodea, Cleoptera, Diptera, and Hymenoptera. The Neighbor-Joining (NJ) method was implemented based on the aligned amino acid sequences with bootstrap method with 1000 replicates using the Molecular Evolutionary Genetics Analysis 11 (MEGA 11) software. Sequence homology analysis revealed 60–70% identity among species within the order Lepidoptera. In contrast, sequence identity between Lepidoptera and species from Orthoptera or Blattodea was less than 70%, whereas homology between Lepidoptera and species from Diptera or Hymenoptera exceeded 70%.

**Table S1-S4**

**Table S1.** Primers used for PCR and qRT-PCR.

| Gene name | Primer | Sequence (5' to 3') |
|---|---|---|
| *BmAWH* | Q-BmAWH-F | ACGGAGGCTCCATTTCATCC |
| | Q-BmAWH-R | GGTCCTGTCCATCGGGATTC |
| *Laccase2* | Q-Laccase2-F | CATCTCCCAGCCATCCCACT |
| | Q-Laccase2-R | CGAACCATCATAAACTCCCGT |
| *iAANAT* | Q-iAANAT-F | TGAATCTCGCCGTCAATCTG |
| | Q-iAANAT-R | GAAACTCCATCGCTCAAGGTAG |
| *yellow* | Q-yellow-F | TCGCTTACTCGTGGGAACAGA |
| | Q-yellow-R | ATAGGAGACGCCGTGATGCC |
| *TH* | Q-TH-F | CGCCTTTCCACACACCTGAACC |
| | Q-TH-R | GGGATGCAAGGCCAATTTCTTGCG |
| *DDC* | Q-DDC-F | GATGAAGACATCCGCAACGGTCTCA |
| | Q-DDC-F | ATCTCCAATTTCGTCAAGAGCGTCG |
| *tan* | Q-tan-F | TCAAACAAACCAAGCAGAGC |
| | Q-tan-F | TCGCCTATATCAGCATCAGC |
| *ebony* | Q-ebony-F | CGTTTATACCGCACAGGAGA |
| | Q-ebony-R | GTAGCATAGGACCACGCACT |
| *FibH* | Q-FibH-F | TCTGTGTCATCTGCTTCATCTCG |
| | Q-FibH-R | TATCCAGGACGAAGTAAGAAACAA |
| *FibL* | Q-FibL-F | ATACCGATTGGTCACATAACAG |
| | Q-FibL-R | GCAGATAGATGGGCGATAA |
| *Fhx* | Q-Fhx-F | TAGGTGGCGTTGAAGTATGG |
| | Q-Fhx-R | AGCCGCTGTGGCAGTTTTG |
| *Sericin1* | Q-Sericin1-F | CACAACCGATAAGACGAG |
| | Q-Sericin1-R | GACGAAGTGGAGGAAGC |
| *Sericin2* | Q-Sericin2-F | CATCGGCTGACTACCA |
| | Q-Sericin2-R | CATCGGCTGACTACCA |
| *Sericin3* | Q-Sericin3-F | TGTCTCGTCGGTGGAA |
| | Q-Sericin3-R | TTGTTGTATGACTGGCTCT |
| *Sericin4* | Q-Sericin4-F | GGCTTAGTTGGCGGTCTCTT |
| | Q-Sericin4-R | GGTTTCATGGTTCACGCTTC |
| *Vg* | Q-Vg-F | AGTCACGACGAATACCAAGAAGAT |
| | Q-Vg-R | TACGATAGTCCTGTGTGAAACG |
| *ESP* | Q-ESP-F | CCGACGACGGCTACCATTTG |
| | Q-ESP-R | GCGAGTGACTTACTGGGACC |
| *30Kc19* | Q-30Kc19-F | ACAGGACTCGACGCTCCAAAG |
| | Q-30Kc19-R | GCTGCTCATTATCATTGGTTCG |
| *VgR* | Q-VgR-F | ACGACAGAGAACAGCAACAG |
| | Q-VgR-R | AAGCAACAGGAGATACCGTG |
| *GSP* | Pig-L-GSP1 | TCATTTTGACTCACGCGGTCGT |
| | Pig-L-GSP2 | CGCATTGACAAGCACGCCTC |
| | Pig-L-GSP3 | AAGCGGCGACTGAGATGTCC |
| *GSP* | Pig-R-GSP1 | GATGAGGATGCTTCTATCAAC |
| | Pig-R-GSP2 | ATGAAGTGCCTGGTACATC |

| Gene name | Primer | Sequence (5' to 3') |
|---|---|---|
| | Pig-R-GSP3 | ATAAGGCGAAAGGCAAATG |
| *WWP* | UN-WWP1 | CGTCTCCAGTCTCCATGTGTTCGTC |
| | UN-WWP2 | CGTCTCCAGTCTTAGGCACAGTGTC |
| | UN-WWP3 | CGTCTCCAGTCTAGTCAGTCAGGTC |
| *Rp49* | Q-Rp49-F | GCATCAATCGGATCGCTATG |
| | Q-Rp49-R | GGACCTTACGGAATCCATTTG |

**Table S2.** List of different insect species used for multiple sequence alignments and phylogenetic tree construction.

| Orders | Species | Accession numbers |
|---|---|---|
| *Lepidoptera* | *Bombyx mori* | NP_001243941.1 |
| *Lepidoptera* | *Melitaea cinxia* | XP_045458163.1 |
| *Lepidoptera* | *Ostrinia nubilalis* | XP_063837923.1 |
| *Lepidoptera* | *Ostrinia furnacalis* | XP_028156698.1 |
| *Lepidoptera* | *Manduca sexta* | XP_030040026.1 |
| *Lepidoptera* | *Pectinophora gossypiella* | XP_049885875.1 |
| *Lepidoptera* | *Cydia amplana* | XP_063372742.1 |
| *Lepidoptera* | *Cydia strobilella* | XP_063546767.1 |
| *Lepidoptera* | *Leguminivora glycinivorella* | XP_048004524.1 |
| *Lepidoptera* | *Cydia pomonella* | XP_061707762.1 |
| *Lepidoptera* | *Danaus plexippus* | XP_032526325.1 |
| *Lepidoptera* | *Choristoneura fumiferana* | XP_073949383.1 |
| *Lepidoptera* | *Colias croceus* | XP_045509885.1 |
| *Lepidoptera* | *Maniola jurtina* | XP_045783816.1 |
| *Lepidoptera* | *Maniola hyperantus* | XP_034839440.1 |
| *Lepidoptera* | *Galleria mellonella* | XP_052752642.1 |
| *Hymenoptera* | *Neodiprion pinetum* | XP_046467649.1 |
| *Hymenoptera* | *Bombus pascuorum* | XP_060815126.1 |
| *Orthoptera* | *Anabrus simplex* | XP_068085154.1 |
| *Blattodea* | *Cryptotermes secundus* | XP_023712209.1 |
| *Diptera* | *Uranotaenia lowii* | XP_055606913.1 |
| *Diptera* | *Drosophila melanogaster* | NP_001261379.1 |
| *Diptera* | *Drosophila elegans* | XP_017127815.1 |
| *Diptera* | *Aedes albopictus* | XP_029735786.2 |
| *Diptera* | *Aedes aegypti* | XP_021699017.1 |
| *Coleoptera* | *Leptinotarsa decemlineata* | XP_074030026.1 |
| *Coleoptera* | *Tribolium madens* | XP_044269750.1 |
| *Coleoptera* | *Tribolium castaneum* | XP_008190523.1 |
| *Coleoptera* | *Zophobas morio* | XP_063911223.1 |
| *Coleoptera* | *Tenebrio molitor* | XP_068901064.1 |

**Table S3.** The quantitative data of cocoon traits of wild type wild-type Dazao (WT) and *BmAWH*-OE lines (female).

| Traits | DZ WT (Mean ± SD) | OE (Mean ± SD) | % Changes (OE vs DZ) |
|---|---|---|---|
| Whole cocoon weight (WCW) (g) | 1.05 ± 0.09 | 0.68 ± 0.07 | 37 |

| | | | |
|---|---|---|---|
| Cocoon shell weight (CSW) (g) | 0.11 ± 0.01 | 0.06 ± 0.01 | 5 |
| Cocoon Shell ratio (CSR) (g) | 0.11 ± 0.01 | 0.09 ± 0.01 | 2 |

**Table S4.** The quantitative data of cocoon traits of wild type wild-type Dazao (WT) and *BmAWH*-OE lines (male).

| Traits | DZ WT (Mean ± SD) | OE (Mean ± SD) | % Change (OE vs DZ) |
|---|---|---|---|
| Whole cocoon weight (WCW) (g) | 0.78 ± 0.05 | 0.48 ± 0.06 | 30 |
| Cocoon shell weight (CSW) (g) | 0.11 ± 0.01 | 0.05 ± 0.01 | 6 |
| Cocoon Shell ratio (CSR) (g) | 0.14 ± 0.01 | 0.10 ± 0.01 | 4 |
